# Applications of OPM-MEG for translational neuroscience: a perspective

**DOI:** 10.1038/s41398-024-03047-y

**Published:** 2024-08-24

**Authors:** Marion Brickwedde, Paul Anders, Andrea A. Kühn, Roxanne Lofredi, Martin Holtkamp, Angela M. Kaindl, Tineke Grent-‘t-Jong, Peter Krüger, Tilmann Sander, Peter J. Uhlhaas

**Affiliations:** 1 Charité-Universitätsmedizin Berlin, Corporate Member of Freie Universität Berlin, Humboldt-Universität Berlin, and Berlin Institute of Health, Department of Child and Adolescent Psychiatry, 13353 Berlin, Germany; 2 Physikalisch-Technische Bundesanstalt, Berlin, Germany; 3 Charité-Universitätsmedizin Berlin, Corporate Member of Freie Universität Berlin, Humboldt-Universität Berlin, and Berlin Institute of Health, Sektion für Bewegungsstörungen und Neuromodulation, Klinik für Neurologie und Experimentelle Neurologie, 10117 Berlin, Germany; 4 Bernstein Center for Computational Neuroscience, Humboldt-Universität, Berlin, Germany; 5 NeuroCure, Exzellenzcluster, Charité-Universitätsmedizin Berlin, Berlin, Germany; 6 DZNE, German center for neurodegenerative diseases, Berlin, Germany; 7 Berlin School of Mind and Brain, Humboldt-Universität zu Berlin, Berlin, Germany; 8 Berlin Institute of Health, Berlin, Germany; 9 Charité-Universitätsmedizin Berlin, Corporate Member of Freie Universität Berlin, Humboldt-Universität Berlin, and Berlin Institute of Health, Department of Neurology, Epilepsy-Center Berlin-Brandenburg, 10117 Berlin, Germany; 10 Charité-Universitätsmedizin Berlin, Corporate Member of Freie Universität Berlin, Humboldt-Universität Berlin, and Berlin Institute of Health, Department of Pediatric Neurology, 13353 Berlin, Germany; 11 Charité- Universitätsmedizin Berlin, Corporate Member of Freie Universität Berlin, Humboldt-Universität Berlin, and Berlin Institute of Health, Center for Chronically Sick Children, 13353 Berlin, Germany; 12 Charité- Universitätsmedizin Berlin, Corporate Member of Freie Universität Berlin, Humboldt-Universität Berlin, and Berlin Institute of Health, Institute of Cell Biology and Neurobiology, 10117 Berlin, Germany; 13 Institute for Neuroscience and Psychology, Glasgow University, Scotland, United Kingdom

**Keywords:** Neuroscience, Diagnostic markers, Pathogenesis, Physiology, Scientific community

## Abstract

Magnetoencephalography (MEG) allows the non-invasive measurement of brain activity at millisecond precision combined with localization of the underlying generators. So far, MEG-systems consisted of superconducting quantum interference devices (SQUIDS), which suffer from several limitations. Recent technological advances, however, have enabled the development of novel MEG-systems based on optically pumped magnetometers (OPMs), offering several advantages over conventional SQUID-MEG systems. Considering potential improvements in the measurement of neuronal signals as well as reduced operating costs, the application of OPM-MEG systems for clinical neuroscience and diagnostic settings is highly promising. Here we provide an overview of the current state-of-the art of OPM-MEG and its unique potential for translational neuroscience. First, we discuss the technological features of OPMs and benchmark OPM-MEG against SQUID-MEG and electroencephalography (EEG), followed by a summary of pioneering studies of OPMs in healthy populations. Key applications of OPM-MEG for the investigation of psychiatric and neurological conditions are then reviewed. Specifically, we suggest novel applications of OPM-MEG for the identification of biomarkers and circuit deficits in schizophrenia, dementias, movement disorders, epilepsy, and neurodevelopmental syndromes (autism spectrum disorder and attention deficit hyperactivity disorder). Finally, we give an outlook of OPM-MEG for translational neuroscience with a focus on remaining methodological and technical challenges.

## Introduction

The search for biomarkers and pathophysiological mechanisms in psychiatry and neurology is a major objective in current research [1]. One important prerequisite for the success of this approach is the widespread availability of non-invasive imaging techniques able to provide assessments of large-scale networks, which can be linked to pre-clinical research [2].

Structural and functional Magnetic Resonance Imaging (MRI/fMRI) techniques have been extensively applied due to their excellent spatial resolution for anatomical and functional networks. However, this approach is subject to several limitations as the measured signals are only indirectly related to the underlying physiology. Moreover, the temporal resolution of fMRI is in the range of seconds. In contrast, large-scale brain networks operate on a millisecond scale with frequencies of up to 200 Hz, which are fundamental for cognitive processes [2, 3] and are involved in the pathophysiology of brain disorders [4].

Due to their excellent temporal resolution, magnetoencephalography (MEG) and electroencephalography (EEG) are ideally suited for the non-invasive identification of neural activity underlying sensory and cognitive processes. Both techniques record summations of electric currents along the dendritic dipoles of large cell ensembles elicited by postsynaptic potentials. In contrast to the electric activity measured with EEG, MEG-measured magnetic fields are hardly affected by differences in tissue [5]. As a consequence, localization of neuronal signal generators is more precise in MEG data as compared to EEG [6–8].

More recently, MEG has been applied to psychiatric and neurological syndromes, such as schizophrenia, autism spectrum disorders (ASDs), dementias, epilepsy, and movement disorders, leading to novel insights in the pathophysiology of cognitive and motor symptoms [5].

Despite these promising results, the application of MEG in translational research is limited, as MEG-systems so far consisted of superconducting quantum interference devices (SQUID-MEG). Such systems are expensive in acquisition as well as maintenance, including extensive cryogenic cooling. In addition, measurements in children are difficult with SQUID-MEG systems due to the rigid position of the sensors in a helmet. Moreover, SQUID-MEG systems do not allow head-movements and thus pose similar constraints for applications in pediatric and clinical populations as MRI/fMRI.

OPM-MEG has the potential to address several shortcomings of conventional SQUID-MEG and EEG systems [9] with significant implications for the application of MEG in basic and translational research. Accordingly, in this paper, we will provide an overview of the current state-of-the-art of OPM-MEG-technology in healthy populations. We will then review potential applications of OPMs for the investigation of psychiatric and neurological conditions. Specifically, we will focus on the relevance of OPM-MEG for the identification of biomarkers and circuit deficits in schizophrenia, movement disorders, dementias, epilepsy as well as neurodevelopmental syndromes.

## Optically pumped magnetometers (OPMs)

**Fig. 1:**
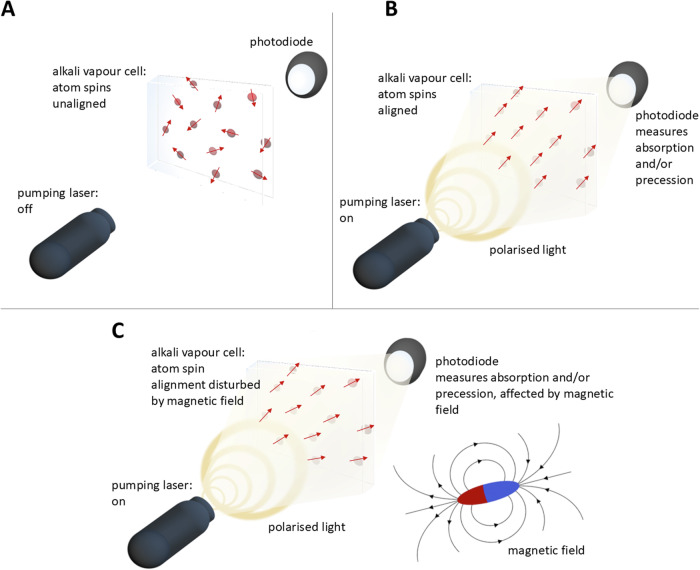
OPM-Methodology. **A** Alkali atoms are moving inside a vapor cell in a thermal random mixture of spin states. **B** ‘pumping’ the vapor cell with a laser producing polarized light induces transitions of most atoms into the same spin state. **C** The amount of light passing through the vapor cell becomes a function of the magnetic field, such as from brain activity, and can be measured with a photodiode.

OPMs consist of vapor cells usually filled with alkali gas (e.g. rubidium, potassium or cesium) or helium. Directing circularly polarized laser light onto the cells results in the atoms in the ensemble to transition to a single quantum state after initially randomly populating many such states. This process, known as optical pumping, leads to the magnetic moments or spins of the atoms to align, so that the ensemble becomes macroscopically magnetized. The light transmission properties of such a state are highly dependent on external magnetic flux density, including that arising from brain activity. In this way, detecting the intensity (or polarization) of light transmitted through the vapor cell (see Fig. 1) can be a sensitive measurement of brain signals (see Shah & Wakai [10], Tierney and colleagues [11] as well as Schofield and colleagues [12] for more in-depth discussion).

**Fig. 2:**
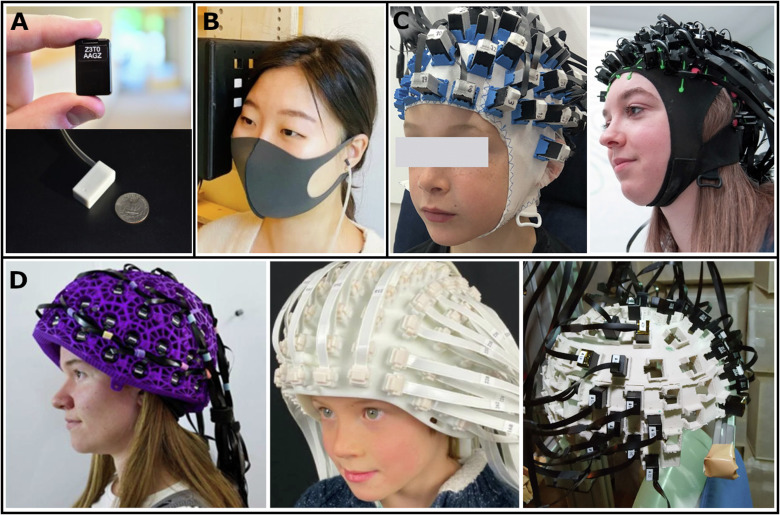
OPM sensors and sensor arrays. **A** Size of current generations of commercially available OPM sensors is as small as a USB stick (Quspin - https://quspin.com; Fieldline - https://fieldlineinc.com) (**B**) OPM sensors can be rigidly installed around a person’s head [146] (**C**), onto caps, similar to EEG electrodes [25] (Quspin - https://quspin.com) or (**D**) fitted into 3-D-printed helmets [147], (10.1111/nyas.14935). All arrangements can be adjusted for individual head sizes. No study participants are displayed in this figure and all material was edited with permission.

In contrast to cryogenic SQUID-MEG systems, OPM-MEG does not require constant helium cooling. Alkali-based OPM sensors internally heat up to ~150 °C to reach optimal operation conditions, translating to temperatures up to ~40 °C on the outside of the sensor casing [11]. OPM sensors based on different gas atoms, such as Helium, can be operated without temperature adjustments, but currently still come at the cost of a higher noise floor [13]. The lack of cooling requirements eliminates the necessity for a rigid, bulky helmet, and enables compact sensors sizes (current commercially available OPMs: ~1.2 × 1.7 × 2.6 cm; see Fig. 2). While SQUID-MEG helmets generally constrain movement, even slight head movements alter the distance and angle between the sensors and the source of brain activity, which introduces errors in the source reconstruction process. The compact sensor sizes of OPMs allow flexible arrangement on the surface of the skin, completely resolving movement-restrictions posed by SQUID-MEG systems. Additionally, flexible placement of OPM sensors introduces new opportunities to record from sites, which are difficult to target with SQUID-MEG and EEG systems [14, 15].

**Fig. 3:**
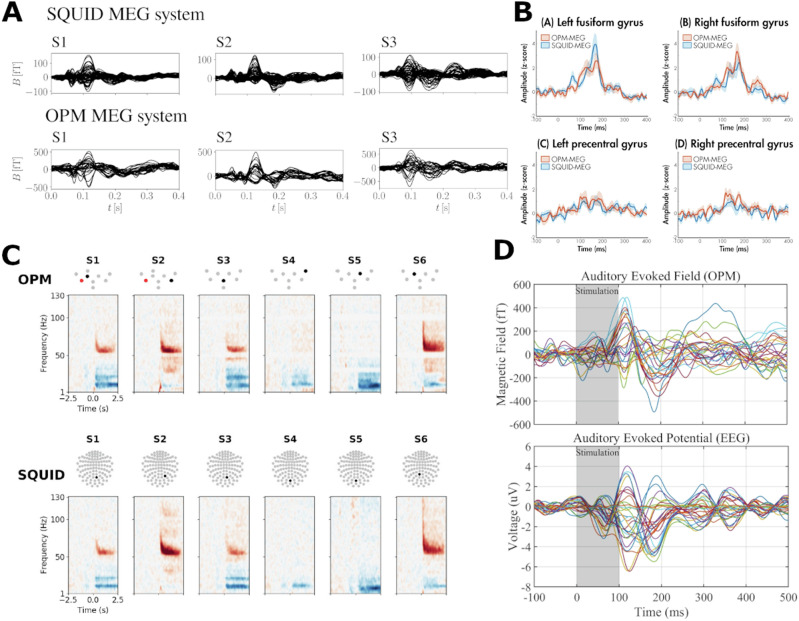
Comparison between OPM, EEG and SQUID-MEG. **A** Comparison between SQUID-MEG and OPM-MEG for auditory evoked fields (n = 3 participants: S1, S2, S3) [30]. **B** Comparison between SQUID-MEG (blue line) and OPM-MEG (red line) for 80 trials of processing emotional (angry or happy) faces of source constructed M170 responses in 15 participants [148]. **C** Comparison of individual OPM-MEG and SQUID-MEG measurements during visual gamma responses for 6 comparable sensors (n = 1) [26]. **D** Comparison of auditory evoked fields (OPM-MEG) and potentials displaying typical differences between EEG and MEG-systems (n = 1) [24]. No changes were made to the original figure material, which was published under OPEN ACCESS license (CC BY 4.0; https://creativecommons.org/licenses/by/4.0/).

Furthermore, the distance of OPM-MEG sensors to the signal source is reduced compared to SQUID-MEG systems. Since the magnetic field strength of dipolar sources decays at the magnitude of the squared distance to the sensor (inverse square law) [16], this leads to increase in signal strength captured by OPM-MEG sensors, especially for cortical sources (see Fig. 3). In some conditions, early accounts estimate the signal strength to be ~ 4–8 times higher than for conventional SQUID-MEG systems [17, 18].

Moreover, the integration of invasive recordings in animals or patients with SQUID-MEG currently presents significant challenges [19–22]. Accordingly, the flexibility of OPM-MEG sensors, which can be placed next to and around such equipment, could facilitate multimodal comparisons, which are crucial to validate and enhance our understanding of the underlying network activity.

## Application properties of OPM-MEG, SQUID-MEG, and EEG devices

Comparisons between OPM-MEG, SQUID-MEG and EEG underly certain caveats, as each recording modality relies on different sensor/electrode properties, especially when comparing EEG and MEG. Nonetheless, it is crucial to evaluate the performance of OPM-MEG in empirical settings compared to the current gold-standard of non-invasive neural time-series imaging, i.e., SQUID-MEG and EEG. Since the innovation cycle for OPMs is still very fast we do not aim for a parametric comparison of modalities but feel an application centered comparison is more informative. An example of how parametric comparison can be rapidly outdated is the recent increase in the linear regime of OPM sensors from ±1 nT to at least ± 50 nT, which strongly impacts the requirements for magnetic shielding. Table 1 summarizes the current status of selected application properties and parameters between EEG, SQUID-MEG, OPM-MEG as well as parallel EEG and OPM-MEG.

**Table 1:** Comparison of application properties between SQUID-MEG, OPM-MEG and EEG.

Parameter	SQUID-MEG	EEG	OPM-MEG	Combined EEG & OPM-MEG	Source
High-frequency oscillation	yes	yes	<130 Hz, but 2000 Hz bandwidth sensor development	Only EEG, but 2000 Hz bandwidth OPMs in development	^130^
Event-related activity	yes	yes	yes	both	^137^
Localization general	yes	yes	yes	improvement expected using both	^29^
Localization deep sources	only tangential sources	yes	under investigation	improvement expected using both	^8^
Localization tangential sources	superior to EEG	yes	superior to EEG	improvement expected using both	^18^
Localization radial sources	no	superior to MEG	under investigation	improvement expected using both	^18^
Spatial resolution	good	bad	very good	improvement expected using both	^38^
Artefacts	medium	medium	under investigation	yes	^138^
Movement	restricted, medium artefacts	possible, strong artefacts	possible, medium artefacts	yes	^138.139^
Connectivity	yes	yes	yes	yes	^41^
Test-retest	yes	yes	yes	yes	^42.140^
Dynamic range	± 20 nT	not limited	± 5 nT (up to ± 150 nT in closed loop)		^141^
Noise-floor	2–3 ft/√ Hz	0.4–2.5 μV/ √ Hz	15 – 30 ft/ √ Hz		^130,142,143^
Shielding	required	not required	required, possibly lightweight/mobile shielding in future		^135.136^
Cost	high acquisition, high maintenance	low aqcuisition, low maintenance	high acquisition, (expected to drop), low maintenance		^144^
Bandwidth	no intrinsic limit	no intrinsic limit	< 130 Hz, but 2000 Hz bandwidth sensor development	OPM-MEG limited. improvement expected in future	^13,26,130,141,145^

### Signal-to-noise ratio

OPM-MEG systems have demonstrated superior SNRs compared to EEG systems when measuring auditory evoked and movement-induced oscillatory activity [23, 24]. Moreover, SNRs of OPM-MEG recordings are comparable and even exceed those of SQUID-MEG recordings, such as during median nerve stimulation, visual gamma-band responses, alpha-band activity and interictal epileptiform discharges [17, 25, 26]. Comparable SNRs were observed during visual stimulation and working memory tasks [27, 28]. Nonetheless, some studies also reported decreased SNRs for OPM-MEG systems when measuring auditory and somatosensory evoked fields [29, 30].

However, it is likely that SNRs of OPM-MEG sensors may be further increased in the future. For instance, SNR-levels of OPM-MEG could benefit from reference OPM sensors to create synthetical gradiometers or increasing OPM sensor size to develop intrinsic gradiometers [29–31], which are estimated to lead to a 10-fold increase in SNR for OPM-MEG sensors [18, 30, 32]. The upcoming commercial availability of whole-head OPM-MEG systems with dense coverage [27, 33, 34] also enables noise suppression techniques such as signal source separation or signal source projection, which will further improve the reduction of interference signals [35]. Many parameters affect SNR, among them sensitivity and bandwidth, which can yet improve for OPM sensors. However, it remains noteworthy that intrinsic constraints of OPM sensors pose a finite limit for such improvements, rendering them unable to reach sensitivity and bandwidth of SQUID-MEG systems [36].

### Spatial resolution

Spatial resolution encompasses different aspects from localization of neuronal signal sources to differentiation between distinct sources in close proximity. Simulations have estimated enhanced performance of OPM-MEG compared to SQUID-MEG systems regarding both of these parameters [18, 32, 37]. Indeed, first empirical evidence confirmed that one OPM-sensor, which was used to sequentially record at 13 different locations, reached localization accuracy for the somatosensory evoked N20 response, which was comparable in SNR to a 174-sensor SQUID-MEG system [17].

Similarly, an 8-OPM-sensor system over the visual cortex localized a focal, grating-induced gamma-band activity with comparable results to a 306-sensor SQUID-MEG system [26]. The spatial specificity of source estimations could likely be improved even further by combining OPM-MEG sensors with EEG electrodes, taking advantage of their complementary features [37]. One important advantage of OPM-MEG compared to SQUID-MEG is the possibility for multiaxial recordings. Some of the latest commercial OPM sensors now feature three sensing directions per sensor, which could substantially increase beamformer source-localization performance. In theory, noise suppression techniques such as signal source separation will also benefit from the triaxial sensor development [38].

Furthermore, OPM-MEG offers novel opportunities for the measurement of neural signals originating from deeper sources, such as the cerebellum and hippocampus [14, 39], which remains challenging for both EEG and SQUID-MEG systems. Combining placement of OPM sensors in the mouth with OPM-sensors above the temporal lobe facilitated the recording of hippocampal brain activity [39]. Similarly, OPM sensors can be placed at the lower back of the head and neck area, improving the accessibility of signals from cerebellum and early visual areas [14].

### Connectivity

Brain disorders are characterized by fundamental changes in functional connectivity that underlie clinical symptoms and cognitive deficits [2, 40]. A recent study tested functional connectivity for the first time applying a 50-OPM-sensor array during rest as well as during a finger abduction and visual task. Analysis of resting-state and task-induced connectomes yielded similar results to a 275-channel SQUID-MEG system [41]. This result was replicated using 56 triaxial OPM sensors, each featuring three channels measuring different directions during presentation of a movie. Connectomes differed between participants but showed high test-retest reliability within participants, comparable to the quality of measures reported with SQUID-MEG systems [42]. A summary of the application properties discussed in the preceding sections and how they compare between systems is given in Table 1.

## OPM-MEG for translational neuroscience

Advances in OPM-MEG technology offer several important applications for translational research, with the potential to significantly advance the understanding and detection of clinical conditions such as schizophrenia, dementias, movement disorders, epilepsy, and developmental disorders.

### Schizophrenia

Schizophrenia represents a severe psychiatric syndrome, characterized by pronounced functional and cognitive impairments [43, 44]. To elucidate the mechanisms underlying cognitive deficits, a substantial body of research applying EEG and MEG has investigated neural oscillations and event-related fields in schizophrenia patients [45, 46].

A consistent finding in this line of research is the reduced amplitude and synchrony of gamma-band (>30 Hz) oscillations [47, 48] in patients with schizophrenia. High-frequency oscillations require GABAergic interneuron-mediated inhibition [3,49] and NMDA-receptor-mediated excitatory drive [50], both of which have been implicated in circuit deficits in schizophrenia [51–54]. In addition to impaired gamma-band oscillations, schizophrenia has also been associated with deficits in low-frequency rhythms, both during resting-state [55] and during task-related activity [56].

Given the converging evidence for the crucial role of rhythmic neuronal activity in the pathophysiology of schizophrenia from pre-clinical [57] and patient studies [46, 47], powerful electrophysiological methods are imperative to further advance insights into the origin of circuit deficits in the disorder. OPM-MEG measurements offer superior signal-to-noise ratios compared to EEG [23, 24] and possibly also to SQUID-MEG [17, 25, 26]. Moreover, the assessment of subcortical activity, such as from hippocampus and cerebellum, which have been implicated in schizophrenia [58, 59], are very challenging to assess using SQUID-MEG and EEG systems. Accordingly, the increased flexibility provided by OPM-MEG systems could facilitate measurement of these structures in patient populations [14, 39].

**Fig. 4:**
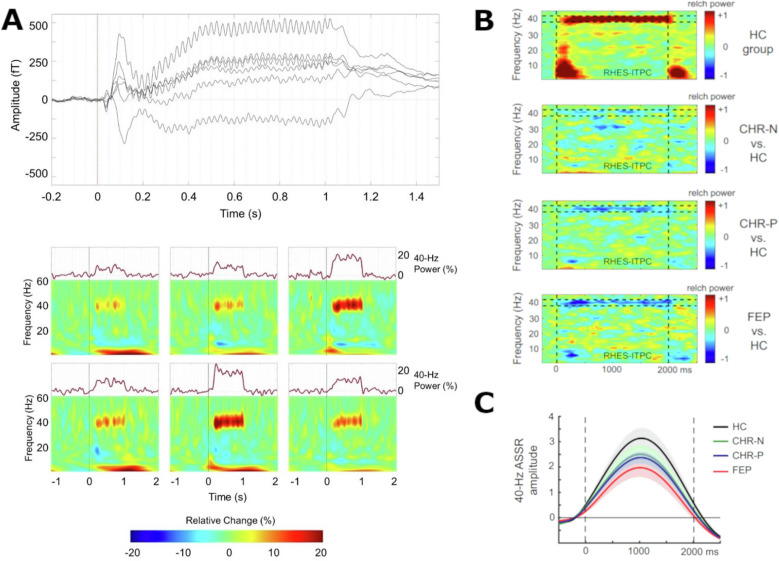
40 Hz auditory steady-state responses during normal brain functioning and emerging psychosis. **A** Average traces of six OPM sensors (top) and their time-frequency-analysis (bottom) over 250 trials [146]. Even without narrowband filtering, the 40 Hz response is clearly visible in each sensor (N = 22 healthy participants). **B** Inter-trial phase coherence, which illustrates the phase synchrony of the 40 Hz response over trials, and the difference of clinical groups from healthy controls (HC = 49 healthy controls; CHR-N = 38 participants with substance abuse and affective disorders; CHR-P = 116 participants with clinical high risk for psychosis; FEP = 33 participants with first episode psychosis). There is no difference between CHR-N and healthy controls, but the differences between CHR-P as well as FEP and healthy controls is apparent [62]. **C** The amplitude of the 40 Hz response in right Heschl’s Gyrus (auditory cortex) for the populations described in **B**. CHR-Ps as well as show a significantly reduced amplitude compared to healthy controls. No changes were made to the original figure material, which was published under OPEN ACCESS license (CC BY 4.0; https://creativecommons.org/licenses/by/4.0/).

Another advantage of OPM-MEG over traditional SQUID-MEG systems lies in its potential for widespread implementation in clinical settings due to reduced operating requirements and cost. This accessibility holds particular significance for research into individuals at clinical high-risk for developing psychosis (CHR-P) [60]. In the majority of cases, the onset of psychosis is preceded by a CHR-P state, characterised by subtle signs and symptoms coupled with functional and neurocognitive impairments. As a result, CHR-P criteria have been established to enable the detection of individuals at an elevated risk of developing a psychotic disorder [60]. Individuals with this diagnosis bear to a cumulative risk of 20% over the course of 2 years [61]. Emerging evidence indicates that neural oscillations in the gamma-band range are impaired in CHR-P participants, constituting a potential biomarker for early detection and diagnosis [62] (see Fig. 4).

Moreover, a widespread availability of OPM-MEG could further advance our understanding of the different illness subtypes, thus allowing stratification and address the considerable heterogeneity of the phenotype. Currently, SQUID-MEG-studies are typically restricted to small patient samples, applied in few selected laboratories around the world. Advances in computational modeling [63] as well as machine learning [64] have enabled the identification of illness subtypes with potentially distinct circuit deficits [65], introducing the possibility for tailored pharmacological and behavioral interventions. Taken together, OPM-MEG not only holds the potential to substantially advance our understanding of circuit deficits in schizophrenia research but could also pose an important milestone in the advancement of a precision psychiatry approach [66].

### Alzheimer’s disease

Alzheimer’s disease (AD) represents the most common form of dementia, affecting approximately 11% of the global population aged 65 years and above [67]. This neurodegenerative condition is associated with a wide range of cognitive impairments, primarily characterized by initial memory deficits, followed by a decline in visuo-spatial and executive processes. The pathophysiology of AD has been linked to alterations in neural oscillations encompassing both low and high frequencies [4]. Specifically, resting-state neural oscillations are characterized by a decrease in spectral power at alpha, beta and delta band frequencies, while the contribution of theta activity to the power spectrum is increased [40]. While, especially during movement, current OPM-MEG systems suffer from increased magnetic interference at slow frequencies such as the theta-band [28], it has been shown that oscillatory activity in this range can be measured with OPM-MEG [28]. Symptom severity and the extent of cognitive deficits have been related to reductions in functional connectivity in the alpha band range [68]. Moreover, evidence from task-related SQUID-MEG-recordings suggests that observed alterations of spontaneous neural oscillations affect functional networks implicated in memory processes [69].

Individuals with mild cognitive impairment (MCI), an age-related neurocognitive disorder that is associated with an increased risk for the development of AD, also exhibit alterations in neural oscillations during cognitive tasks as well as at rest [70–72]. Given the progressive nature of cognitive and circuit deficits in AD, early identification and stratification of individuals with MCI is a question of major clinical and scientific importance [73]. Preliminary evidence suggests that resting-state MEG-data may serve as a potential biomarker for the early detection of this initial illness stage [74].

However, the application of SQUID-MEG faces limitations due to the advanced age and pronounced cognitive and motor symptoms exhibited by AD-patients, for example. Consequently, acquiring measurements requiring prolonged periods without movement poses considerable challenges. By contrast, OPM-systems have demonstrated to provide reliable data during movement [25, 75], which could significantly facilitate measurements in larger AD-cohorts.

### Movement disorders

Neurodegenerative movement disorders, including Parkinson’s disease (PD) and dystonia, are characterized by progressive motor symptoms such as tremors, rigidity and abnormal postures with a chronic often debilitating course [76]. A common observation in these syndromes refers to aberrant oscillatory activity primarily detected in the basal ganglia through intracerebral deep brain stimulation (DBS) electrodes [77–80]. The utilization of this unique invasive approach to accessing deep brain structures has facilitated the development of the concept that movement disorders should be regarded as network disorders. Specifically, alterations in oscillatory basal ganglia activity serve as indicators of symptom patterns rather than disease-specific spectral patterns.

Increased beta oscillations (13-35 Hz) have been associated with hypokinetic symptoms, such as slowness of movement [81, 82], while increased low frequency (3–12 Hz) [83–85] and gamma activity (60–90 Hz) [86] have been linked to hyperkinetic symptoms, such as involuntary movements or muscle contractions. At a network level, parallel recordings of whole-head SQUID-MEG and intracerebral recordings via DBS-electrodes have enabled the characterization of physiological oscillatory patterns during voluntary movements across the motor circuit [20, 21] and disentangled spectral features of distinct cortex-basal ganglia pathways in PD patients [87, 88]. Oscillatory correlates of PD symptoms, however, have yielded less conclusive results when employing non-invasive recording techniques, such as SQUID-MEG or EEG [89–91]. Here, enhanced SNR in parallel recordings of ECoG and DBS-electrodes have shed light on the increased phase-amplitude coupling between subcortical beta and cortical gamma oscillations [92]. In dystonia patients, parallel whole-head SQUID-MEG and DBS-recordings have indeed revealed a symptom-related decrease of alpha coherence (9-12 Hz) between the cerebellum and the internal pallidum, which is the output nucleus of the basal ganglia [22].

Neuroimaging studies have emphasized the cerebellum’s crucial role in the etiology of several movement disorders and its potential as target structure for therapeutic neuromodulation [93–95]. The difficulty to investigate cerebellar activity using SQUID-MEG and EEG poses a significant roadblock in this regard, which OPM-MEG systems might help overcome.

Beyond motor symptoms, sensory symptoms occur in over 70% of patients with movement disorders. These can range from paresthesia to auditory impairments or even visual hallucinations [96]. Through flexible sensor placement, such as at the lower back of the head, and close proximity to the signal source, OPMs hold the potential to improve recordings from sensory cortices such as visual cortices [14] as well as deep auditory cortices. This advantage of OPMs could significantly expand our understanding of the relatively unexplored role of the sensory system in movement disorders.

Taken together, OPMs could advance research and diagnostic measurements in patients afflicted by movement disorders, providing access to brain areas that are difficult to assess with conventional EEG and SQUID-MEG systems. Furthermore, the limitations imposed by involuntary movements, particularly tremors, severely compromise the applicability and data quality of EEG and SQUID-MEG recordings. In contrast, OPMs offer to provide high-quality assessments even during such involuntary movements.

### Neurodevelopmental disorders

Autism spectrum disorder (ASD) and attention deficit hyperactivity disorder (ADHD) are two prominent neurodevelopmental syndromes with a typical onset around 1 to 2 years for ASD [97] and 2 to 7 years for ADHD [98]. Similar to schizophrenia, disturbances in the balance between excitation and inhibition have been implicated in circuit deficits and altered neural oscillations in ASD [49, 99]. This is consistent with the view that schizophrenia and ASD are neurodevelopmental disorders, albeit with distinct developmental trajectories, fundamentally involving disturbances in the maturation of cellular parameters related to effective neuronal inhibition [100]. Neural oscillations in both children and adults with ASD are impaired, in particular at alpha and gamma-band frequencies [101–103], where reductions in spectral power [104, 105] as well as functional connectivity [106] have been demonstrated. Early detection and intervention have been shown to improve the trajectory of children with ASD [107] and there is preliminary evidence that neural oscillations are altered in children with familial risk for the development of ASD [108].

In ADHD, altered neural oscillations particularly in the alpha-band have been investigated in the context of attentional processes, which are a prominent sign of the disorder [109]. Oscillatory alpha activity is involved in attention and perception processes, thought to prioritise the processing of relevant over irrelevant information [110–112]. In ADHD, aberrant modulation and lateralization of alpha activity has been reported across development [113, 114] and impairments in event-related alpha desynchronization during visual selective attention have been observed [109].

OPMs may offer several applications for the investigation and diagnosis for ASD and ADHD. Firstly, movements cause severe acquisition problems both in SQUID-MEG as well as EEG. In addition, there are significant variations in head sizes during development. This poses a significant challenge for SQUID-MEG-measurements in both healthy as well as clinical developmental populations. The adaptability of sensor arrangements to individual head-shapes and sizes of OPM-MEG systems together with a reduction in movement-induced artefacts and increased SNR compared to EEG could dramatically enhance the application of OPM-MEG in children with ADHD and ASDs.

Finally, OPM-MEG could facilitate research of impairments in social cognition, which are an essential aspect of the clinical presentation and diagnostic criteria of ASD [115, 116]. The novel method of hyperscanning applies simultaneous measurements of brain activity in two individuals [117]. However, fMRI as well as SQUID-MEG allow hyperscanning experiments only via video, without in-person interaction. OPM-MEG systems could potentially overcome these shortcomings as recently demonstrated [118], which could be harnessed to research the origin of altered social interactions in ASD and other syndromes.

### Epilepsy

Epilepsy is one of the most common neurologic diseases and affects more than 50 million people world-wide. Unfortunately, one third of all patients with epilepsy continue to have seizures despite treatment with at least two adequately administered antiseizure medications [119]. Epilepsy in these patients is referred to as drug-resistant epilepsy, since the chance of seizure-freedom through further antiseizure medication is as low as <5% [120, 121]. These patients call for evaluation of potential epilepsy surgery, since a surgical removal of a seizure focus on a subset of patients with focal-onset seizures renders up to 70% of them seizure-free [122].

Surgery planning and assessment is, however, more complicated in patients who lack a lesion in a structural 3 Tesla MRI. Some of these individuals therefore require more in-depth evaluation including in some cases invasive EEG recordings to identify the epileptic focus and to further delineate eloquent cortical structures that need to be preserved. Many of these invasive approaches bear a significant risk of complications, in particular hemorrhage [123].

Interictal MEG is complementary to scalp EEG recordings and may provide new and clinically relevant information in these individuals. A large study of 1,000 consecutive patients with drug-resistant epilepsy demonstrated that in 32% of cases with focal-onset seizures MEG yielded additional helpful information to the findings of existing presurgical non-invasive procedures [124]. Thus, MEG in presurgical evaluation may prevent the need for intracranial EEG or contribute to planning of the exact placement of intracranial electrodes; overall, its use results in higher rates of post-operative seizure freedom.

OPM-MEG may offer several advantages over current presurgical evaluation as measurements allow for head-movements and even locomotion during recordings [25], which is of particular importance for young children and individuals with intellectual deficit and/or behavioral problems. A first study applying OPM-MEG in children suffering from epilepsy showed that a 32-sensor system could detect interictal spikes with higher SNR and comparable localization accuracy compared to a 204-sensor SQUID-MEG system [25]. Moreover, other studies showed that OPM-MEG can reliably detect interictal epileptiform discharges, further validated by intracerebral (stereotactic) EEG recordings [125–128].

In summary, in patients with epilepsy, OPM-MEG could enhance the localization of the epileptic focus, possibly removing the necessity for invasive recordings. Furthermore, the versatility of OPM systems extends their utility to pediatric populations and enables measurements during movement, thereby broadening their applicability to patient groups that were previously ineligible for such assessments.

## Summary and outlook

OPM-MEG constitutes a novel technology for non-invasive neuroimaging, providing several major advantages over conventional SQUID-MEG and EEG systems. Given the importance of temporal signatures of brain activity for the understanding of psychiatric and neurological conditions [4], we believe that a wide availability of OPM-MEG systems in basic research as well as clinical settings could significantly advance insights into the origin of circuit deficits as well as support the development of biomarkers.

Moreover, the rapid development and optimization of OPM-MEG technology promises further improvements in the future. To this end, several OPM-sensors have been introduced and tested, which differ in bandwidth, sensitivity, noise characteristics, dynamic range and temporal response. For instance, adaptations to cell sizes in current-generation OPM-sensors are likely to improve SNRs further, while the introduction of whole-head OPM-MEG systems will increase the spatial resolution of source estimation procedures [129]. Finally, recent advancements in sensor development utilizing helium-cells overcame the limited bandwidth (<~200 Hz) of previous generations [130]. Unlike rubidium-cell sensors that require heating for optimal performance, helium sensors operate heating-free and feature estimated bandwidths of up to 2000 Hz.

As an alternative approach to combining a growing number of commercial or research-level individual OPM sensors into arrays, scalable high-density OPMs in miniaturized and integrated assemblies could be used as magnetic imaging chips or cameras [131]. This type of approach addresses multiple challenges present in current systems: (1) A higher sensing density of <10 mm sensor-sensor spacing enables more accurate source localization. (2) By construction, an integrated chip device features precise relative sensor placement, which is an essential prerequisite in source localization and (3) an ab initio multi-sensor design avoids technical problems such as heat management and inter-sensor crosstalk.

Despite the significant progress made, several challenges remain in order to fully exploit the potential of OPM-MEG for translational research. One such challenge is the relatively high noise floor. Although the close proximity to the signal source partially compensates for this aspect, achieving a reduction in the noise floor comparable to the state-of-the-art in SQUID-MEG systems would further enhance the signal-to-noise ratio of OPMs [9].

In addition, it should be noted that, similar to SQUID-MEG and most fMRI systems, OPM-MEG systems require a magnetically shielded environment for optimal performance. While sensor developments also explore the possibility of shielding-free applications [132], it is uncertain whether OPMs will provide reliable recordings of brain activity in the presence of large magnetic background fields [33, 133, 134]. However, due to their relatively small size, OPM-MEG systems can be operated in a more cost-effective, compact and lightweight shielding environment, which can be further improved with active field compensation [135, 136].

In conclusion, a wide distribution of OPM-MEG systems in basic and clinical research promises the possibility to advance insights into the origin and diagnosis of psychiatric and neurological disorders. We believe that these novel developments will be beneficial for research into neurodevelopmental syndromes such as ADHD and ASD, forms of dementia, movement disorders, epilepsy and schizophrenia. Taken together, OPM-MEG could be an important method for translational neuroscience that could alleviate the considerable burden of disease associated with these syndromes through improved diagnosis and also potentially novel therapies.
